# How Ethnoracial Groups Spend Their Time

**DOI:** 10.7758/rsf.2025.11.1.09

**Published:** 2025-01

**Authors:** SARAH JAMES, ELIZABETH WRIGLEY-FIELD

**Affiliations:** Demographic and Decennial Research Group of the Center for Economic Studies at the U.S. Census Bureau, United States.; Department of Sociology and the Minnesota Population Center at the University of Minnesota, United States.

**Keywords:** race-ethnicity, time use, u-index, unpleasant experience, emotions, leisure

## Abstract

We know strikingly little about how time use varies across ethnoracial groups in the United States. We describe the daily lives of 210,586 White, Black, Hispanic, and Asian people in the nationally representative American Time Use Survey (2003–2019). Activities are similarly unpleasant for all groups, but White people spend the most time on highly pleasant leisure activities, Asian people spend the most time in unpleasant ways, and Black people spend the most time doing affectively neutral activities, such as watching television. These patterns show continuity in across recent decades and in harmonized historic data. Black people spend the most and Hispanic people the least time alone. We conclude that time diaries are a promising resource for exploring nuances in the texture of ethnoracial groups’ daily experiences.

How do people in the United States spend their time? The broad contours of most adults’ lives are similar: they spend most waking hours of most days of the week doing paid work (and sometimes also work toward formal schooling); sleep for many hours a day; and divide the remainder among travel, leisure, caregiving, and other forms of household labor. At the population level, time use patterns are dominated by activities that take large amounts of time on many days per week, occur repeatedly, and typically involve fixed commitments lasting many years—for example, engaging in paid work or raising children. These activities play such an outsized role in structuring daily time use that even relatively limited variation in them (for example, in the portion of a subpopulation that has young children) can produce meaningful differences in time use in the aggregate. On the other hand, smaller variations in daily schedules on the margins of those major fixed activities can also add up to meaningful differences in the textures of people’s daily lives. For example, variations in how people spend their leisure time typically affect a smaller portion of each day than time doing paid work, but these variations add up to meaningful differences when they are summed across days and across groups. Both types of variation are a product of constraints—in material needs, neighborhood characteristics, and the availability and needs of others, to name a few—and of the choices that people make amid these constraints.

It is striking that there is little systematic analysis of time use for distinct racial groups in the United States. Chadwick Curtis, Julio Garín, and Robert Lester (2022) come closest, measuring racial disparities in a theoretical model of overall utility. Other studies “control for” race without truly analyzing it. Recently, scholars of health (Gee et al. 2019) and of racism (Kwate 2017) have noted the clear need for such analyses, and others have developed a theoretical model of how differences in time use and control over time may structure racial disparities in health outcomes (Colen et al. 2023). Yet a recent systematic review of time use research never mentions race or racism (Cornwell, Gershuny, and Sullivan 2019). This gap is surprising: in the United States, racial stratification structures patterns of daily life through pathways like access to economic opportunities, the physical and social geographies of residential neighborhoods, and household structures. For example, in the New York metro area, recent decades have seen increases in the share of White adults with short commutes to high-paying jobs and the share of Black and Hispanic adults with long commutes to low-wage jobs (McLafferty and Preston 2019). These variations in life experience may inform distinctive preferences, goals, and constraints across groups. Yet we do not know how ethnoracial differences in time use patterns combine to produce differences in daily life across ethnoracial groups or whether these differences are stable.

How much time do ethnoracial groups spend on core activities such as work, housework, and leisure? With whom do they spend their time? In what contexts do they carry out these activities? How do people feel while doing them? Perhaps most important, how do activities and emotions combine to produce inequities in quality of life? We argue that how people spend their time and the way they feel during these daily activities generates important differences in typical experiences for different racial groups, a consequential domain of racial stratification that has been too little studied. To address these limitations, we use the nationally representative American Time Use Survey to lay out the contours of what 210,586 White, Black, Hispanic, and Asian people did each day in the 2000s and 2010s, who they did it with, where they did it, and how they felt while it was happening. Then we use the American Heritage Time Use Survey to situate contemporary disparities in patterns going back to 1965.

In the course of this analysis, we establish several new stylized facts about aspects of daily life in the United States. Our results reveal continuity in daily experiences in the 2010s compared to earlier time periods. These patterns, though long-standing, have not been explored previously. We find important differences in time use across groups. For example, White people spend the most time on highly pleasant leisure activities, Asian people spend the most time in unpleasant ways, and Black people spend the most time doing affectively neutral activities, such as watching television. Black people also spend the most, and Hispanic people the least, time alone. These findings indicate that time use is, and has long been, a fundamental aspect of racial differences in life experiences in the United States.

## BACKGROUND

The last half century has seen economic and cultural shifts in how Americans spend their time. For example, from the 1960s to the 2000s, leisure time became more available to less-educated Americans but also declined in quality (Sevilla, Gimenez-Nadal, and Gershuny 2012). In recent decades, researchers have also documented a growing “time squeeze” of increasing conflict between the time demands of work and of other elements of life. This shift has occurred across national context with changes in working hours and the life-course timing of carework obligations (Ford et al. 2021).

Despite these broad narratives about changes in Americans’ time use, we know little about whether these changes have been similar for members of different racial groups. The dearth of research in this area is particularly striking in comparison to the robust field of research on gender differences in time use since the mid-twentieth century. Although women’s and men’s time use has become more similar since the mid-1960s (Bianchi et al. 2012; Sayer 2016), daily unpleasantness decreased from 1965 to 2005 for men only (Krueger 2007).^1^ Over the same period, civil rights movements restructured American institutions of daily life but racial stratification has persisted and reemerged in new forms (Derenoncourt et al. 2022; Katz, Stern, and Fader 2005; Logan 2013; Reardon and Owens 2014). These changes are certainly as substantial as the concurrent shift in gender norms, but research has largely neglected exploring whether or how ethnoracial groups’ daily experiences have changed over time. Indeed, given these countervailing trends, it is unclear whether to expect convergence or divergence in daily life across ethnoracial groups. A few studies of gender differences do consider race—for example, the gender gap in housework is smallest for Black couples and largest for Hispanic couples (Sayer and Fine 2011)—but we have limited information about differences beyond highly gendered activities.

### Pathways Suggesting Ethnoracial Differences in Time Use

There are several reasons to expect systematic differences in how racial groups spend their time.

First, time is structured by employment in ways that differ for ethnoracial groups. Labor-force participation rates (Groshen and Holzer 2021), paid work time (Perry-Jenkins and Gerstel 2020), and work schedules (McCrate 2012) all vary across ethnoracial groups. Outsized rewards to long work hours have increased in recent decades (Weeden, Cha, and Bucca 2016), which may exacerbate racial pay gaps in a manner similar to their role in gender pay gaps (Goldin 2014).

Second, residential racial segregation affects how ethnoracial groups spend their time. For example, Black and Hispanic people spend more time traveling to shop or work because they live in neighborhoods with fewer retail stores (Schuetz, Kolko, and Meltzer 2012; Zenk et al. 2005) that lack nearby economic opportunities (McLafferty and Preston 2019). These differences are exacerbated by use of slow public transportation (Kwate 2017; Holt and Vinopal 2023). Additionally, differences in housing stock, neighborhood amenities, and perceived public safety create differences in time spent indoors versus outdoors, such as in green space (Kephart 2022) and in public versus in private spaces (Kwate 2017).

Third, ethnoracial groups differ in the type and timing of major demographic events such as family formation and mortality, criminal justice system involvement, and patterns of coresidence. These differences mean that ethnoracial groups have different networks of people to spend time with across the life course. For example, Black people are more likely than White people to live alone (Park, Sheen, and Clark 2025). Differences in patterns of coresidence also have implications for carework across ethnoracial groups: Hispanic women are most likely to live with children, Black women have the highest rates of coresidence with dependent elders before midlife, and Black and Hispanic women are more than twice as likely than White women to live with grandchildren at midlife (Ice 2023).

Fourth, administrative burdens impose substantial losses of time, as well as leading people to be unable to access services and benefits to which they are entitled (Herd and Moynihan 2019; Herd et al. 2023; Jackson 2020). Recent analyses show that low-income people spend substantially more time waiting to access basic services than high-income people do, and high-income Black people wait as long as low-income people, aggregated across racial groups (Holt and Vinopal 2023).

Fifth, chronic exposure to stressors changes people’s preferences on how they spend their time due to overactivation of the stress response system (Friedman et al. 2017). Relative to their White counterparts, Black and Hispanic adults experience higher levels of overall stress, acute life events, relationship stressors, financial stressors, neighborhood violence and disorder, and discrimination (Sternthal, Slopen, and Williams 2011). Even though Black people report more psychological distress, less life satisfaction, and lower happiness than White people, Black people also report fewer depressive or anxious systems, fewer psychiatric disorders, and higher levels of flourishing (Williams 2018)—the so-called Black-White mental health paradox (Brown, Mitchell, and Ailshire 2020; Thomas Tobin et al. 2022). The psychosocial stress of being a member of a minoritized group may also contribute to differences in how people choose to spend their time. For example, the effortful hypervigilance of managing (typically White people’s) perceptions during daily activities (Williams 2018) may cause minoritized groups to change their daily activities to minimize this stress (Hornbuckle 2021): Black men are less likely to do physical activity in neighborhoods they perceive to be whiter (Ray 2017), and Black and Hispanic adults who experience discrimination report high levels of attention to their appearance to minimize negative interactions (American Psychological Association 2016). Yet research on daily experiences among minoritized groups has been limited to examining researcher-defined lists of experiences that are assumed to be stressful for all people or stressors that occur in particular domains (such as discrimination at work). The omission of smaller daily activities from these lists means that these estimates miss important details.

### Contribution

In sum, we know surprisingly little about the broader contours of time use across racial groups, despite strong evidence that pathways such as residential racial segregation, social and kin networks, varied preferences, and psychosocial stress would cause differences. Likewise, existing evidence of inequities in ethnoracial groups’ daily affect considers only selected experiences, meaning that we do not understand total differences across groups. To address these limitations, we proceed in three steps. First, we use nationally representative time diary data from the American Time Use Survey to describe continuity and change in daily time use across the 2000s and 2010s. Then we consider the emotions that people felt during these daily activities. Across this work, we compare the daily lives of White, Black, Hispanic, and Asian people, extending research that has largely focused on White-Black differences and rarely included Asian people. Finally, we explore how daily experiences differ across ethnoracial groups in the 2000s relative to the 2010s. We situate these contemporary patterns of daily affect to trends in daily unpleasantness since 1965, using the American Heritage Time Use Survey. We conclude by contextualizing these findings in prior research, discussing the strengths and weaknesses of our approach, and offering future directions for this work.

## WHAT PEOPLE DO EACH DAY

To describe patterns of time use, we analyze 4.4 million daily activities of 210,586 people who completed the American Time Use Survey (ATUS) between 2003 and 2019. ATUS is a nationally representative time use study of the noninstitutionalized civilian population collected annually by the Bureau of Labor Statics among a subset of participants in the Current Population Survey age fifteen and older. In a telephone interview, respondents completed a twenty-four-hour recall time diary of their activities from 4 a.m. the prior day to 4 a.m. on the interview day. We obtained ATUS data from IPUMS Time Use at the University of Minnesota (Hofferth et al. 2022). Table 1 describes this analytic sample (column 1), as well as the datasets we use in other parts of the analysis. Online appendix A shows the demographic characteristics of each analytic sample.^2^

### Measures

Time diary respondents describe their daily activities in an open-ended manner, and researchers assign these activities to various activity codes. To ensure that our analyses are comparable with the datasets we analyze elsewhere in this article, we use the seventy-six categories of daily activities harmonized by the Centre for Time Use Research at the University College London. These categories include all activities reported in all surveys. We further collapse these seventy-six activities into sixty-four activities with sufficient sample size to examine ethnoracial group differences.

**Figure 1.**
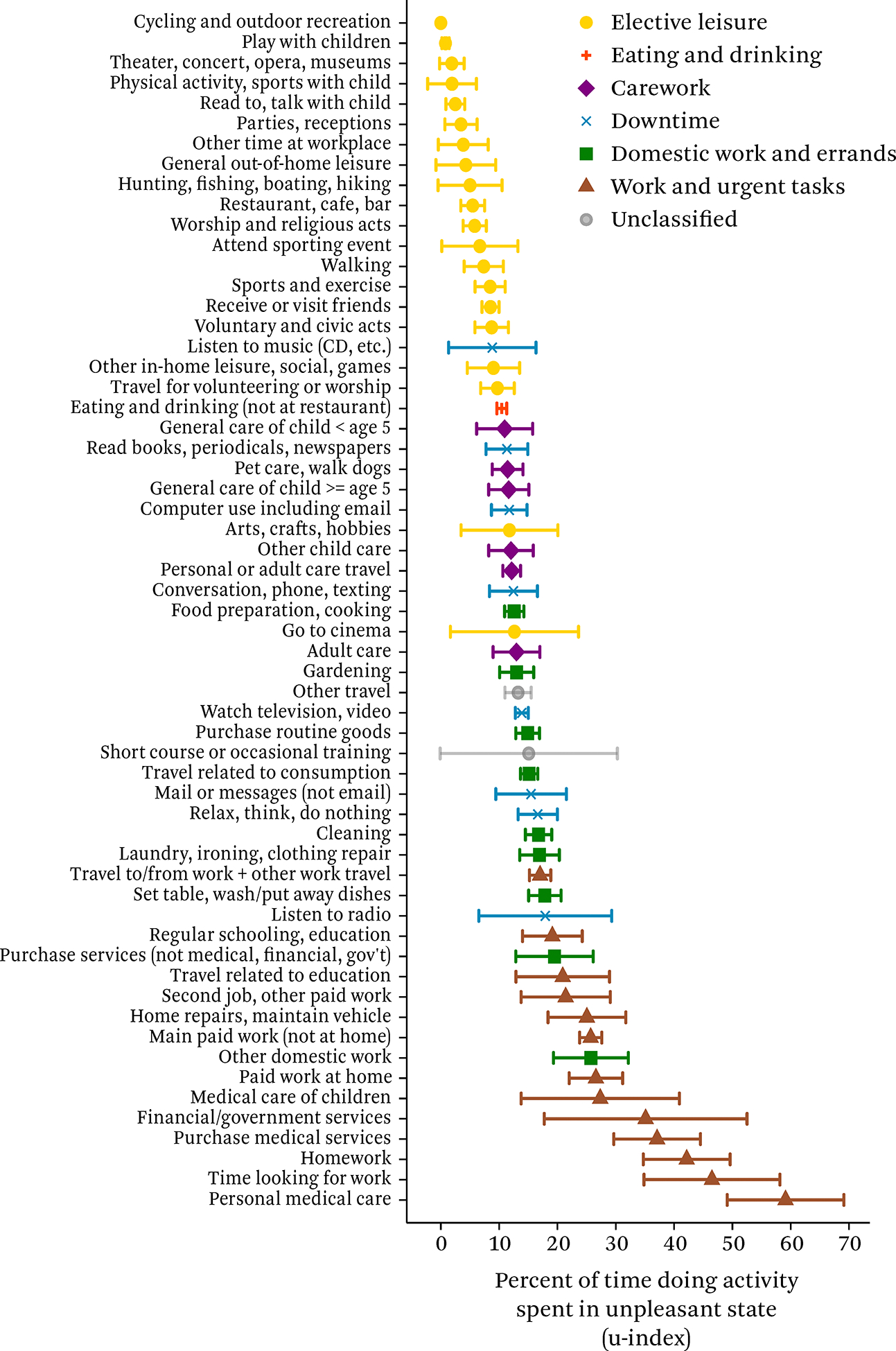
Unpleasantness of Daily Activities *Source:* Authors’ calculations based on American Time Use Survey Well-Being Model, 2010, 2012, 2013 (Flood et al. 2023). *Note:* Estimates and 95 percent confidence intervals. Average u-index values across 160 race-specific u-index calculations (using 160 replicate weights). Confidence intervals represent the uncertainty in the u-index estimate from using 160 different replicate weights.

To simplify our presentation of time spent in daily activities, we group the sixty-four daily activities into six groups (see figure 1). We developed these groups inductively, considering both which activities have similar levels of unpleasantness (discussed later) and the content of the activities. We consider elective leisure activities, eating and drinking, carework, neutral downtime, domestic work, work or urgent tasks, sleep, and personal care time. We borrow the term “neutral downtime” from Alan Krueger (2007) for its evocative terminology but include a slightly different set of activities. As in prior research, neutral downtime is dominated by watching television, which makes up two-thirds to three quarters of neutral downtime for all groups (see online appendix B).

We also consider where activities took place and with whom they were done. Respondents report where activities occurred for activities other than sleep or personal care. We measure whether activities were done in private spaces (own or others’ homes) or at one’s own home. We analyze with whom people did daily activities other than sleep, personal care, and work (data on with whom respondents work is available after 2010 only). We consider several types of people with whom respondents spent their time: no one (time alone), with a spouse or romantic partner, with a child under age five, with a child under age eighteen (including a child under age five), with a coresident child, with extended family (such as parents, grandparents, or cousins), and with friends.

Ethnoracial group includes categories for non-Hispanic White, non-Hispanic Black, Hispanic, non-Hispanic Asian, and other ethnoracial group (including American Indian, Alaskan Native, Hawaiian, Pacific Islander, and multiracial).^3^ We do not show results for the final group because of its small size and heterogeneity. For brevity, we omit the modifier non-Hispanic in the presentation of results.

We present unadjusted and adjusted estimates. Adjusted estimates account for sex (male, female), age, age squared, educational attainment (less than high school education, high school graduate or GED, some college education, college graduate, and any postgraduate education), employment status (full-time employment, part-time employment, not working but not retired, retired), nativity (native or foreign born), marital status (married; widowed, separated, or divorced; or never married), number of children under age eighteen residing in the respondent’s household, urban (defined as living in a Census-designated metropolitan area, reference) versus rural residence, Census region (Northeast, South, Midwest, West), ten-year group (2003–2009 [referred to as 2000s in the presentation of results] and 2010–2019 [referred to as 2010s in the presentation of results]), and the month and day of the week about which the time diary was collected.

### Analytic Strategy

We use count models to describe the activities on which ethnoracial groups spent their time. We model the number of minutes spent on various types of activities, where these activities took place, and with whom they occurred. The distribution of minutes in all activities was overdispersed, with some activities having an excess of zeros. Thus we used negative binomial models to model number of minutes spent on sleep, personal care, elective leisure, eating and drinking, neutral downtime, domestic work and errands, at home, in private spaces, alone, with extended family, and with friends and zero-inflated negative binomial models to model the number of minutes spent on work and urgent tasks, with a spouse or partner, with children under age five, with children under age eighteen, and with a coresident child.

### Results: Daily Activities

#### Differ by Race and Ethnicity

Each ethnoracial group’s time use is distinctive in its own way, though most differences are small. Everyone’s time is constrained by the rhythms of daily life (sleeping, caring for oneself and others, some form of work or functional tasks, and leisure time). As in many other key population-level outcomes (Permanyer, Sasson, and Villavicencio 2023; Salganik et al. 2020), variation within groups is much more substantial than variation across groups, yet the variation across groups is socially meaningful. In the case of time use, small aggregate differences can reflect differences in the (relatively small) portion of each racial group that has a very different pattern of time use than others (such as the proportion with very few work hours) or, sometimes, differences that are relatively few minutes but potentially consequential to daily experience (such as the difference between fifteen minutes or fifty minutes of weekday commuting time).

**Figure 2.**
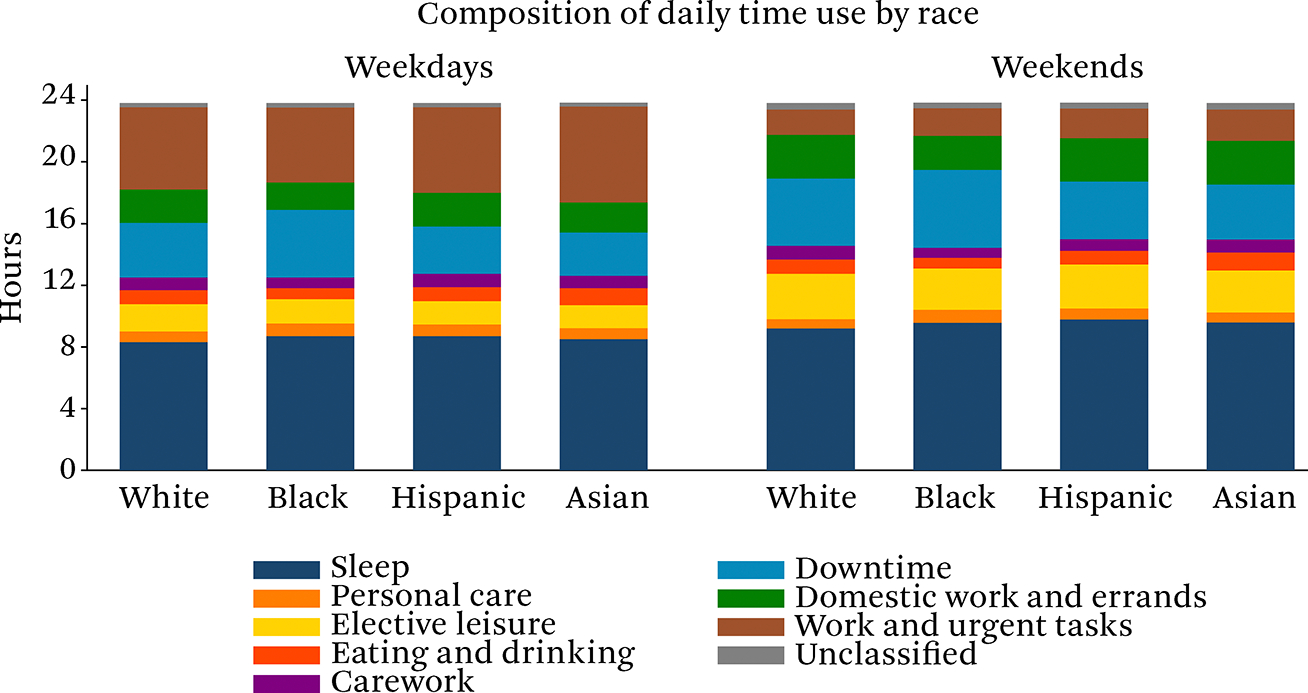
Daily Activities by Race/Ethnicity on Weekdays and Weekends *Source:* Authors’ calculations based on American Time Use Survey 2003–2019 (Flood et al. 2023). *Note:* Weighted, unadjusted descriptive values of time use across all sampled activities on weekdays and weekends from the American Time Use Survey 2003–2019.

Figure 2 shows the overall pattern of daily time use for White, Black, Hispanic, and Asian people on weekdays and weekends. These allocations underscore that meaningful differences—such as Black Americans having the most downtime and the least time spent on work and other urgent tasks, and Asian Americans showing the reverse—occur in the context of broadly shared patterns, such a cadence in which sleep, leisure, and domestic work expand on weekends, when paid work time contracts.

**Figure 3.**
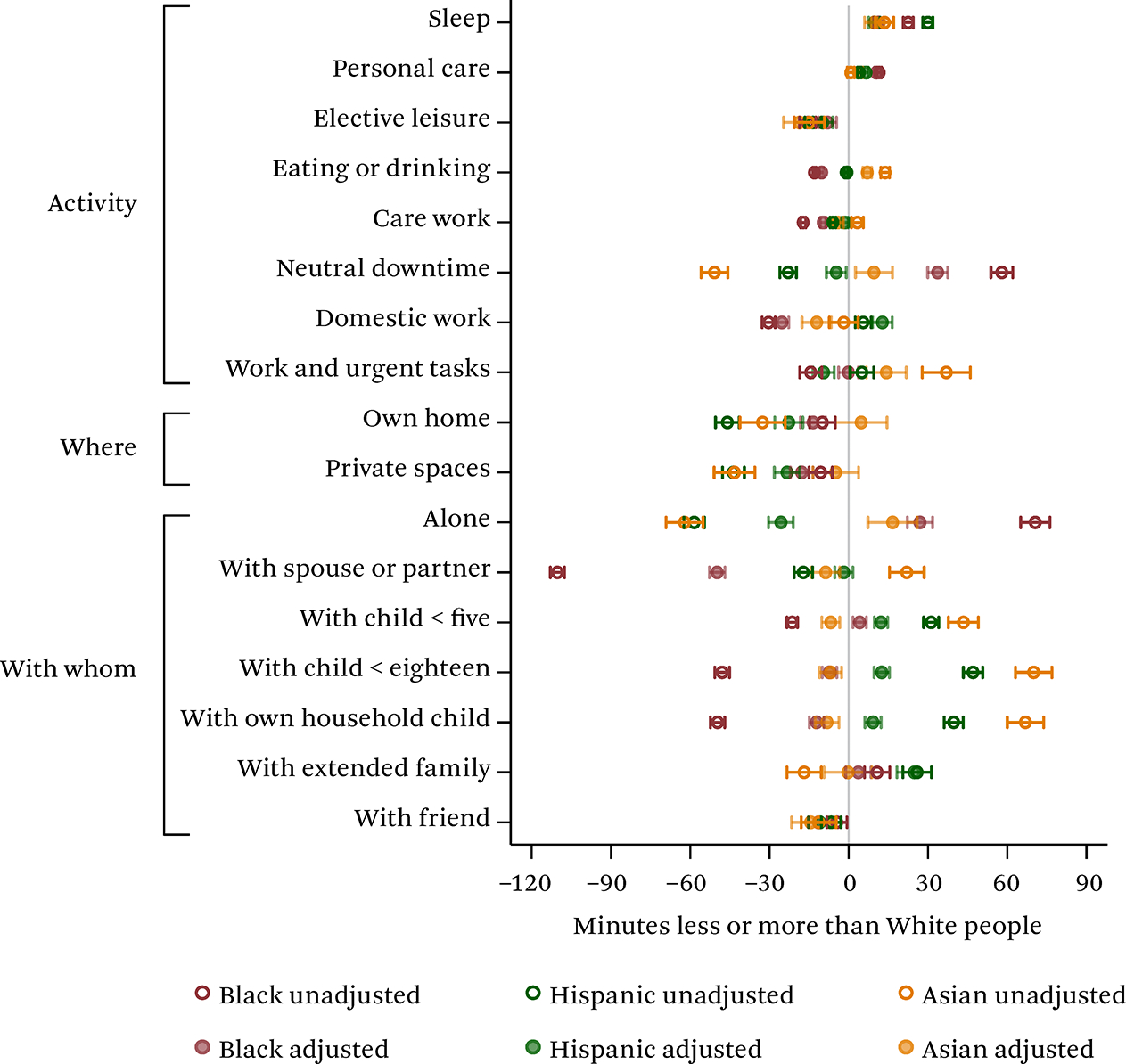
Ethnoracial Differences in Time Spent on Daily Activities *Source:* Authors’ calculations based on American Time Use Survey 2003–2019 (Flood et al. 2023). *Note:* Unadjusted and adjusted predicted values and 95 percent confidence intervals from the American Time Use Survey 2003–2019. Authors’ calculations of marginal effects from count models of time spent on daily activities: negative binomial models of number of minutes spent on sleep, personal care, elective leisure, eating and drinking, neutral downtime, domestic work and errands, at home, in private spaces, alone, with extended family, and with friends and zero-inflated negative binomial models to model the number of minutes spent on work and urgent tasks, with spouse or partner, with children under age five, with children under age eighteen, and with a coresident child. Estimates are adjusted for sex, age, age squared, educational attainment, employment status, nativity, marital status, any coresident children under age eighteen, urban-rural residence, census region, ten-year group, month, and day of week.

Figure 3 shows how Black, Hispanic, and Asian people’s time spent on a given activity per day compares with White people’s in the same activity; we use White people as a baseline because they are the largest group. Online appendix C transforms these values, presenting them as ratios relative to White people’s time use. We show both unadjusted measures (open shapes) and adjusted measures (shaded shapes). These two sets of estimates convey different information. The unadjusted rates show the total differences in time use across ethnoracial groups, which are created by large structural differences across groups (such as level of labor-force participation) as well as individual preferences. These unadjusted estimates speak most directly to the question of how time is experienced differently across ethnoracial groups. The adjusted rates account for a robust (but imperfect) set of covariates related to these structural differences, with the goal of estimating differences in time use between people whose circumstances are broadly similar but whose ethnoracial identity differs. These adjusted estimates—and specifically, differences across ethnoracial groups in these estimates—address whether ethnoracial differences in time use are solely driven by, for example, differences in family structure or employment.

White people spend the most time doing the most pleasant elective leisure activities, in private spaces, and with friends and sleep slightly less than minoritized groups (figure 3). Additionally, White people have the most fragmented time, doing 3 to 10 percent more activities per day than other racial-ethnic groups. Adjusted number of daily activities and 95 percent confidence intervals are White 18.9 (18.8, 18.9), Black 17.2 (17.2, 17.3), Hispanic 18.2 (18.1, 18.3), Asian 18.3 (18.2, 18.5).

Black people spend more time on neutral downtime, less time with a spouse or partner, and more time alone than White people. Black people spend fifty-eight minutes more each day on neutral downtime than White people (unadjusted, figure 3), about 25 percent more than White people, because they do fewer of both highly pleasant and highly unpleasant activities.

**Figure 4.**
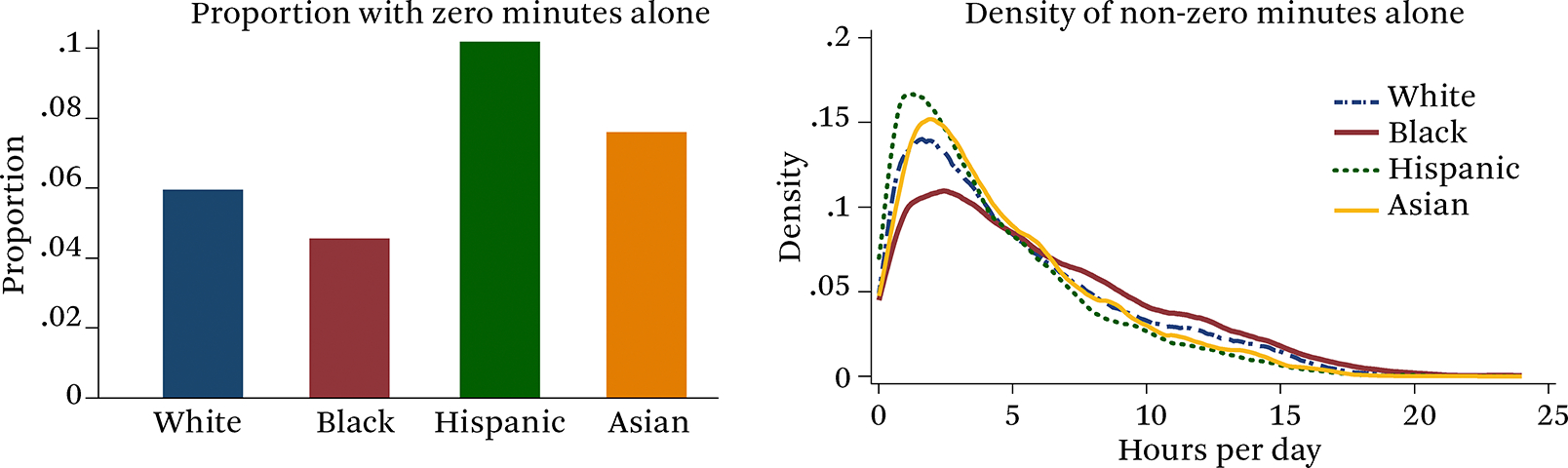
Ethnoracial Differences in Time Spent Alone *Source:* Authors’ calculations based on American Time Use Survey 2003–2019 (Flood et al. 2023). *Note:* American Time Use Survey 2003–2019. Weighted, unadjusted descriptive values of time spent alone across all sampled activities.

Whom Black people spend time with also differs from White people. Black people spend less than half as much time with a spouse or partner each day than White people (unadjusted, figure 3), a difference of an hour and fifty minutes. For context, this large disparity is similar in magnitude to the hour and forty-three minute per day decline in housework that women have experienced since 1965 (Bianchi et al. 2000). Black people do not make up much of this difference by spending time with other family members or friends: Black people also spend the most time alone of any ethnoracial group, spending, on average, nearly five hours (294 minutes) alone each day—or one hour eleven minutes more time alone each day than White people (unadjusted, figure 3), about 23 percent more time alone than White people). These differences persist across the spectrum of alone time (figure 4): Black people are both substantially less likely than other racial groups to spend no time alone on average (fewer than 5 percent of Black people compared with, at the other extreme, 10 percent of Hispanic people) and are also more likely than any other ethnoracial group to spend the bulk of their waking hours alone. These large differences in time alone are striking and admit to different interpretations, as we elaborate on in the discussion.

Although a portion of disparities in neutral downtime, alone time, and time with spouse or partner reflect differences in household sociodemographic characteristics such as household composition and labor-force status—for example, that Black people are more likely than White people to live alone (Park, Sheen, and Clark 2025)—accounting for these factors does not fully explain these differences. In adjusted results (figure 3), relative to White people, Black people have thirty-four more minutes per day of neutral downtime (15 percent more than White people), spend fifty minutes less with a spouse or partner each day (75 percent as much time as White people), and spend twenty-seven more minutes per day alone (9 percent more than White people). Additionally, Black people spend about 29 percent more time on personal care, about 19 percent less time eating or drinking, and 16 percent less time doing domestic work each day than White people do (adjusted).

Hispanic people spend more time with children and with extended family members than White people, and than all other groups after adjusting for covariates, consequently spending less time alone (figure 3). Before adjusting for sociodemographic covariates, Hispanic people spend thirty-one more minutes per day with children under age five (49 percent more time), twenty-six minutes more with extended family (31 percent more time), and fifty-eight minutes less alone (19 percent less time) than White people. Time with children is time that respondent’s main activity—such as eating dinner—is done with a child. It does not include secondary childcare such as supervising a child while cooking dinner. Although a portion of these differences are explained by household and demographic characteristics, differences remain even after adjusting for covariates. In adjusted estimates, Hispanic people spend 19 percent more time with young children, 29 percent more time with extended family, and 8 percent less time alone than White people (see online appendix C).

Asian people work about fourteen minutes more per day and spend about seven minutes more per day eating and drinking than White people (adjusted values, figure 3). Additionally, before accounting for demographic covariates, Asian people spend fifty-one minutes less on neutral downtime, and with somewhat less of this neutral downtime being television watching than for other groups (online appendix B); thirty-two minutes less in their own homes; forty-three minutes less in private spaces; and sixty-two minutes less alone each day than White people due to spending more time with spouse or partner and children (figure 3). The compositional factors accounted for in our demographic covariates fully account for differences in neutral downtime, time in own home, and time in private spaces. Difference in time with others are reversed and have substantially smaller magnitudes after adjusting for covariates. Adjusted estimates indicate that Asian people spend seventeen fewer minutes per day alone than White people do (figure 3).

## HOW PEOPLE FEEL DURING DAILY ACTIVITIES

In 2010, 2012, and 2013, a subset of ATUS participants were selected to participate in a Well-Being Module that collected information on emotional states during daily activities. Over these years, 37,088 people were asked about the degree to which they were happy, in pain, sad, stressed, tired, or found meaning during three randomly selected daily activities. We use data on emotional states during 102,796 daily activities with nonmissing information across emotion measures. We pool data from these three survey years (U.S. Bureau of Labor Statistics 2017).

### Measures

Respondents who participated in the Well-Being Module reported the degree to which they felt happy, in pain, sad, stressed, tired, or found meaning during three randomly selected daily activities using a Likert scale ranging from 0 (not at all) to 6 (very).

We measure the unpleasantness of sixty-four daily activities using the u-index (Kahneman and Krueger 2006), a measure of the proportion of time spent by a population in an unpleasant state. The u-index has the advantage of measuring emotions in a way that is comparable across people who use different ranges of the available Likert scale. It considers which emotion a given person rated most strongly rather than the absolute level of how strongly that emotion was rated. We calculate the u-index for the population of the United States using respondents’ reports of being happy, in pain, sad, and stressed during daily activities. This set of emotions has been used in similar prior work (Krueger 2007). First, we identify whether a respondent’s strongest reported emotion during a given activity was strictly negative, that is, whether the emotion for which they endorsed the highest rating was negative (pain, sad, or stressed). Fifteen percent of activities are rated as unpleasant. Then we produce population-level estimates of the share of time spent in an unpleasant state while doing the activity, as specified by the Bureau of Labor Statistics (2014, 5–7). We calculate these rates separately by ethnoracial group when showing race-specific u-indices (online appendix D).

Specifically, we estimate the population average level of unpleasantness that all Americans experienced during their eligible activities^4^ during a day ($\overset{‾}{U}$) as follows:

(1) $$\overset{‾}{U}=\frac{\Sigma_{i}\Sigma_{k}w_{ik}U_{ik}}{\Sigma_{i}\Sigma_{k}w_{ik}}$$

In this equation, i is the respondent, k is the sampled activity, U is whether the activity is unpleasant, and $w_{ik}$ is the survey weight for activity k for respondent i. We repeat this series of calculations 161 times, once using the nationally representative pooled survey weights from the primary ATUS Well-Being Module and again for each of the 160 replicate weights. Then we average these estimates and calculate their standard deviations across the replicates to produce the final population-level u-index values and their associated 95 percent confidence intervals (figure 1).

### Analytic Strategy

We describe which activities have higher and lower levels of unpleasantness and how these values differ by race. We also analyze ethnoracial differences in rates of reporting only positive emotions during all sampled activities (a binary indicator) using logit models. All analyses include the sociodemographic covariates described previously (see “What People Do Each Day”).

### Results: Daily Emotions Differ Across Groups

We begin by exploring which activities are more unpleasant than others (figure 1). We underscore that these measures are population-level measures of total unpleasantness in the entire population during a given activity, not individual-level analyses. From least to most unpleasant, they are as follows:

- Elective leisure activities, such as recreation, religious activities, time with friends, and exercise, are rated as least unpleasant. Among total time spent on these activities in the United States population, these activities are for the most part are unpleasant less than 10 percent of the time. Several aspects of childcare have very low unpleasantness (such as playing, reading, talking to, or doing sports with children), likely because these are respondents’ primary activities only.
- Eating and drinking, not at a restaurant, is unpleasant about 10 percent of the time. Because eating and drinking are biological necessities, we treat them as a distinct activity.
- Carework includes child, adult, and pet care. Ten to 14 percent of this time is unpleasant.
- Neutral downtime is a diverse group of less-pleasant leisure activities that are for the most part passive activities: watching television and videos (the bulk of the category), listening to music, reading, using computers, conversation, and relaxing.^5^ Activities in this category have high variability in their unpleasantness, ranging from spending about 10 to near 20 percent of time doing the activity in an unpleasant state.
- Domestic work and errands include household tasks such as cooking, gardening, shopping, and laundry. Between 12 and 25 percent of the time spent doing these activities is unpleasant.
- Work and urgent tasks are the most unpleasant daily activities. This group includes both tasks that are routinely scheduled (work, commuting, education) or likely urgent (home repair, medical care, homework, looking for work). These activities are typically unpleasant more than 20, and up to 60, percent of the time.
- Two activities are not classified into any affect-activity group. Other travel includes all other travel not related to specific activities otherwise defined, so we could not determine the purpose of this travel. Short course or occasional training could include a wide range of activities that are either elective (such as a photography course) or obligatory (such as job training).

In general, activities whose timing is imposed by others are rated as more unpleasant than activities whose scheduling is chosen. One example that illustrates this pattern is listening to music: listening to chosen music using a CD-ROM or other device is unpleasant less than 10 percent of the time, whereas listening to the radio (as one’s primary activity) is unpleasant nearly 20 percent of the time. These estimates have overlapping confidence intervals, but the point estimates illustrate the broader pattern.

We do not identify systematic differences in the unpleasantness of daily activities by ethnoracial group. Online appendix D shows the race-specific unpleasantness several exemplar activities.

**Figure 5.**
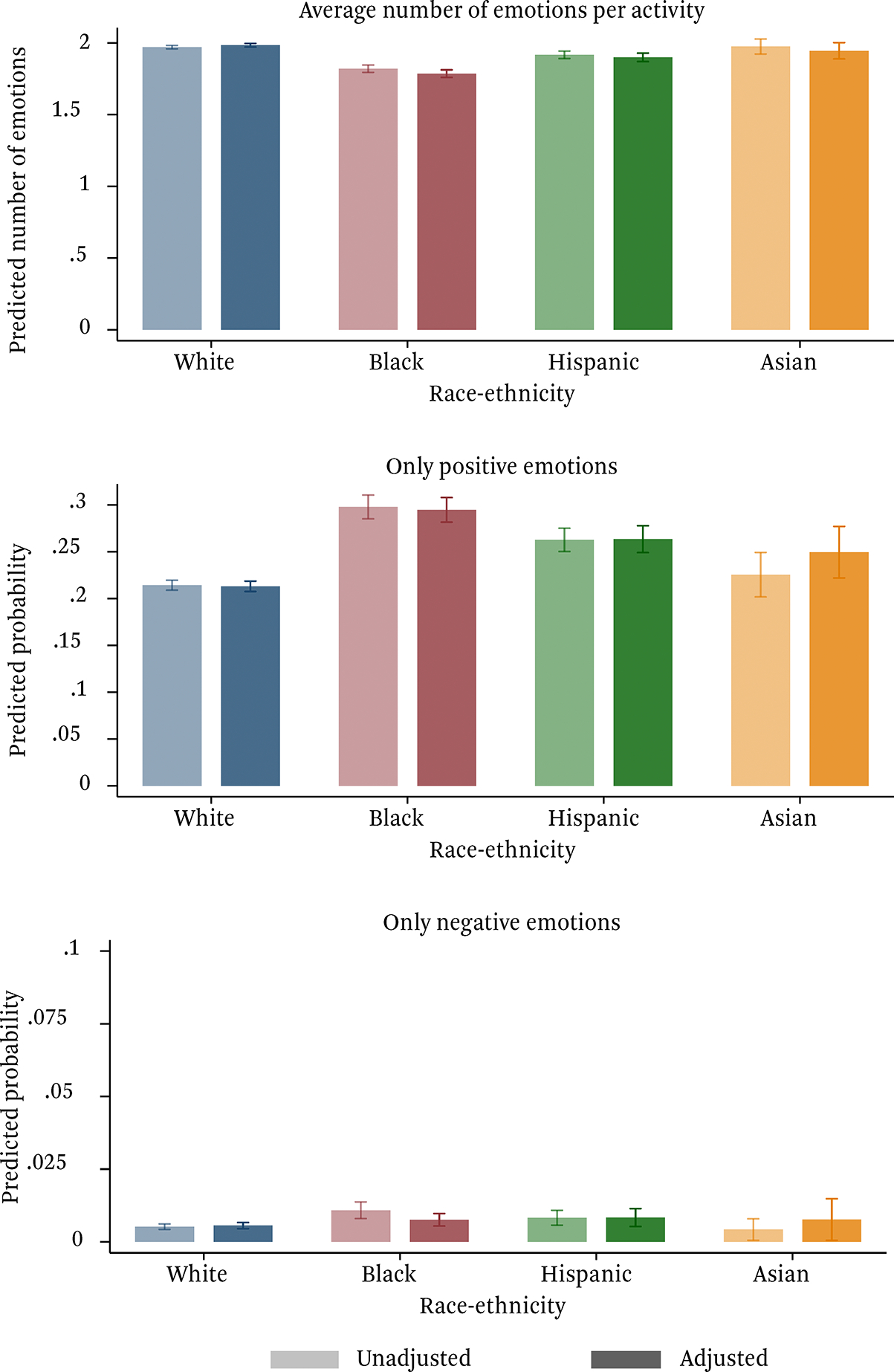
Ethnoracial Differences in Emotions *Source:* Authors’ calculations based on American Time Use Survey Well-Being Model, 2010, 2012, 2013 (Flood et al. 2023). *Note:* Unadjusted and adjusted estimates and 95 percent confidence intervals from the American Time Use Survey Well-Being Model, 2010, 2012, 2013.

However, Black and Hispanic people report fewer emotions per activity than White people (figure 5, top panel). Minority groups are more likely to report only positive emotions across all sampled activities (figure 5, middle panel). It is not clear whether this pattern indicates avoidance of negative emotions or a preference for reporting fewer emotions: ATUS asks about only one positive emotion, happiness, which is also the most frequently reported emotion. Similarly, Black and Hispanic people are more likely to report only negative emotions across all sampled activities (about 1 percent of the sample, figure 5, bottom panel). Rates of reporting only positive emotions in all activities are highest for Black people (figure 5, middle panel) and are consistent across sex, age, and employment levels (not shown).

These findings on reported emotions are perhaps consistent with the Black-White mental health paradox, whereby Black people have more stressful experiences but report better mental health than White people (Brown, Mitchell, and Ailshire 2020; Thomas Tobin et al. 2022; Williams 2018). Consistent with this paradox, people of color are more likely to report only positive emotions during daily activities than White people. However, Black and Hispanic people are also more likely to report only negative emotions during their activities, though this was rare for all groups. These differences occur in part because people of color reported fewer emotions during activities than White people did, suggesting greater within-group heterogeneity in emotional experience.

## HISTORICAL AND CONTEXTUAL VARIATION

We continue to use data on contemporary time use from ATUS and the ATUS Well-Being Module. To add historic context, we add data from the American Heritage Time Use Survey (AHTUS), a harmonized series of time use surveys spanning 1930 to 2018. Studies were harmonized by the Centre for Time Use Research at the University College London and are available through IPUMS Time Use at the University of Minnesota (Fisher et al. 2018). We analyze data from all people age eighteen and older collected in years for which information on respondents’ race is available: 1965–1966 (Multinational Comparative Time-Budget Research Project), 1975 (American’s [*sic*] Use of Time: Time Use in Economic and Social Accounts), 1992–1994 (National Human Activity Pattern Survey), 1994–1995 (National Time-Diary Study), 1998–2001 (Family Interaction, Social Capital, and Trends in Time Use Study and National Survey of Parents), and 2003–2012, 2018 (American Time Use Survey). In total, we analyze the daily unpleasantness of 155,891 people who completed time diaries between 1965 and 2018.

### Measures

Analyses of ATUS and the ATUS Well-Being Module use the same set of measures described earlier. Because the historic AHTUS data is harmonized with these contemporary data, we are able to use a similar set of covariates in our historic analyses. Sex, age, age squared, educational attainment, employment status, number of coresident children under age eighteen, census region, month in which the time diary was completed, and the day of week about which the diary was collected are measured the same way as in the ATUS. Three measures are unavailable in the historic data (nativity, urban-rural residence, and whether people who are not working are retired), and three more are slightly different due to historic data limitations. In the historic AHTUS, consistent information on race at all time periods is limited to White, Black, and Other Race (not specified). We compare all White and all Black respondents at each period, omitting the Other group due to its small size and heterogeneity. In AHTUS, partnership status indicates whether the respondent was currently married or unmarried. Covariates for the time trend include 1960s (1965–1966), 1970s (1975), 1990s (1992–1994, 1994–1995, 1998–2001), 2000s (2003–2009), and 2010s (2010–2012, 2018).

### Analytic Strategy

To describe differences in daily unpleasantness by ethnoracial group, we calculate the share of each person’s day spent in an unpleasant state. Specifically, we apply the population average u-index score described earlier to each person’s mix of daily activities in the larger 2003–2019 ATUS sample (or, for historic analyses, the larger 1965–2018 AHTUS sample).^6^ Analytically, we multiply the time spent in each activity by its u-index (the population average proportion of time spent in an unpleasant state during that activity), sum across all activities to get total minutes spent in an unpleasant state, and divide by the length of the waking day (the individual’s total time spent on activities other than sleep and personal care).^7^ Then we model group differences in the proportion of the waking day spent in an unpleasant state using ordinary least squares regressions with the sociodemographic covariates described previously. In analyses of change over time, we interact ethnoracial group with ten-year period.

We emphasize that we use the same u-index values for everyone in the population because we did not find differences in the unpleasantness of particular activities across groups. Thus differences in daily unpleasantness reflect the time each group spends on activities and does not indicate that members of a particular group find an activity to be more or less unpleasant.

To aid in interpreting the results to follow, we offer an example of how to interpret a difference in daily unpleasantness. Spending an additional 1 percent of the waking day in an unpleasant state is about ten minutes of unpleasant time, presuming a sixteen-hour waking day that allocates eight hours for sleep and personal care. These 9.6 minutes of unpleasantness might come from fifteen additional minutes seeking medical care, unpleasant 66 percent of the time; thirty additional minutes doing paid work, unpleasant 32 percent of the time; or forty-two minutes cleaning, unpleasant 23 percent of the time (see figure 1).

### Results: Continuity of Ethnoracial Differences in Daily Life

**Figure 6.**
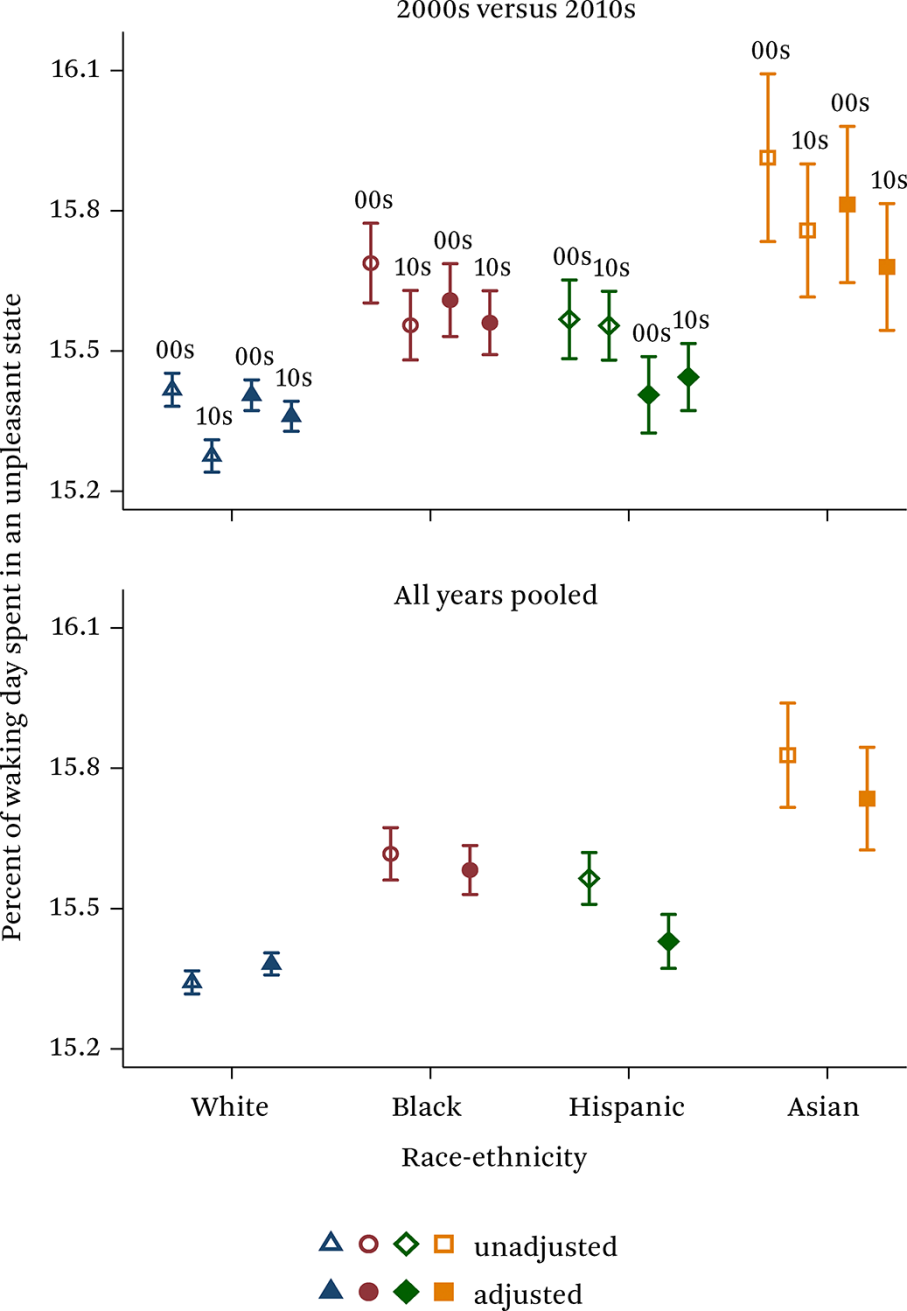
Daily Unpleasantness by Ethnoracial Group *Source:* Authors’ calculations based on American Time Use Survey, 2003–2019 (Flood et al. 2023). *Note:* Unadjusted and adjusted predicted values and 95 percent confidence intervals from the American Time Use Survey, 2003–2019. Predicted level of daily unpleasantness from ordinal least squares regressions of the proportion of the waking day spent in an unpleasant state. Estimates are adjusted for sex, age, age squared, educational attainment, employment status, nativity, marital status, any coresident children under age eighteen, urban-rural residence, census region, ten-year group, month, and day of week. Models comparing the 2000s and 2010s include an interaction term for ethnoracial group × ten-year group.

Total daily unpleasantness is higher for minoritized groups than for White people (figure 6, bottom panel). Unadjusted estimates reveal higher daily unpleasantness for Black, Hispanic, and Asian people compared to White people. Adjusting for sociodemographic characteristics, Black and Asian people have higher daily unpleasantness than White people. Across specifications, Asian people spend the largest share of their waking days in an unpleasant state. Disparities in daily affect for minoritized groups relative to White people are driven by differences in daily activities, because people of all groups rate the same activities as similarly unpleasant (see “How People Feel During Daily Activities”).

Inequities in unpleasant time have remained stable across the 2000s and 2010s (figure 6, top panel). Minoritized groups’ unpleasant time does not vary significantly between the 2000s and the 2010s. White people’s daily unpleasantness is slightly higher in the 2000s than in the 2010s before adjusting for covariates, but these differences are explained by population composition.

**Figure 7.**
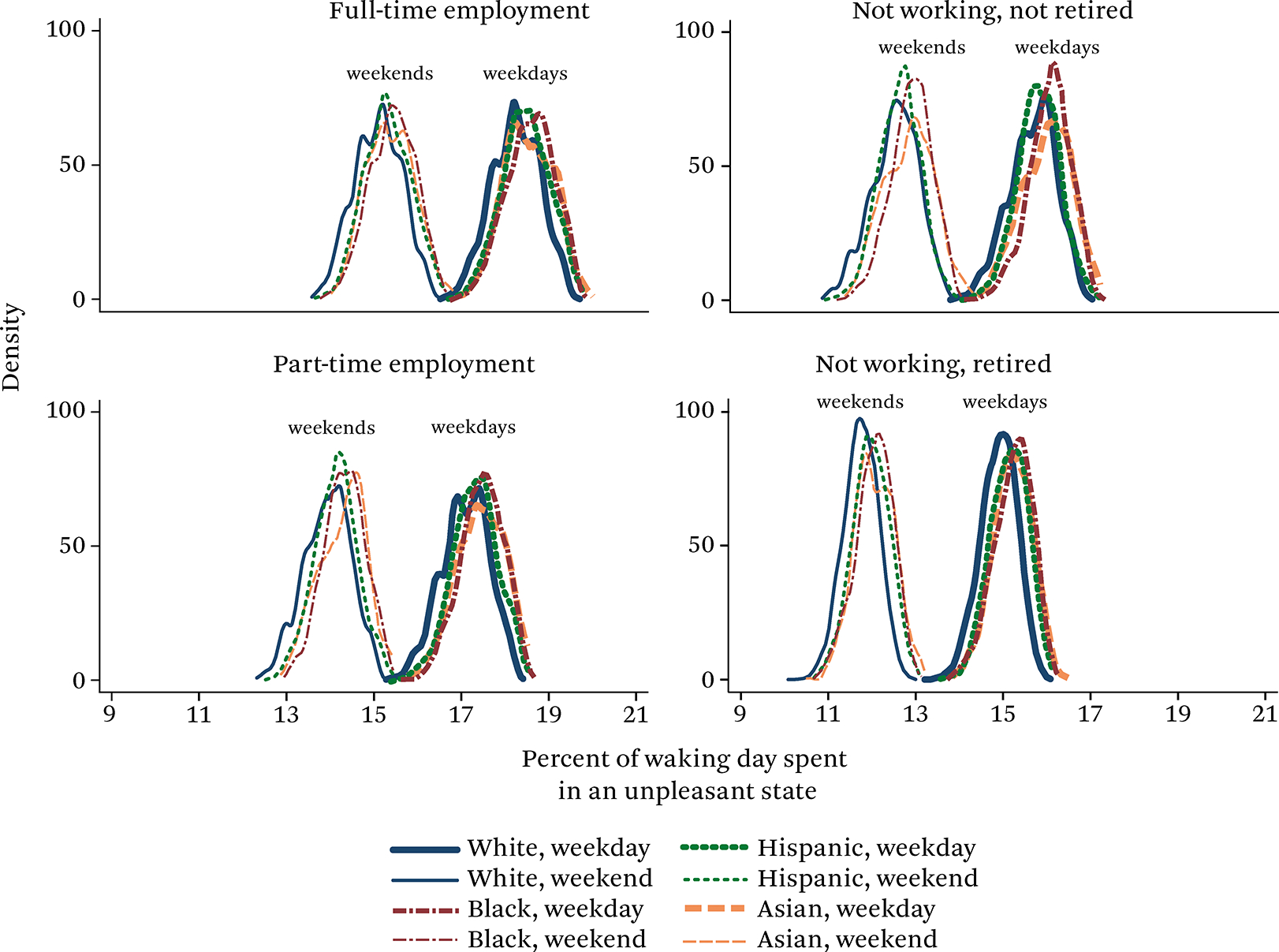
Distribution of Daily Unpleasantness by Ethnoracial Group and Employment Status *Source:* Authors’ calculations based on American Time Use Survey 2003–2019 (Flood et al. 2023). *Note:* Adjusted predicted values from the American Time Use Survey 2003–2019. Authors’ calculations of kernel density estimates of the predicted level of daily unpleasantness from ordinal least squares regressions of the proportion of the waking day spent in an unpleasant state. Estimates are adjusted for sex, age, age squared, educational attainment, employment status, nativity, marital status, any coresident children under age eighteen, urban-rural residence, census region, ten-year group, month, and day of week.

To better understand these patterns, we also compare the distribution of daily unpleasantness by ethnoracial group across employment levels (figure 7). These distributions have two peaks because the weekday-weekend structure provides a distinctive cadence to unpleasant time for all ethnoracial groups: weekdays (thick lines) are more unpleasant than weekends (thin lines), as the composition of activities that people do each day differs on weekdays and weekends. Most people have more choice in how they spend their weekend time than their weekday time. Employed people’s weekends have a similar level of unpleasantness as the weekdays of retired people. Yet figure 7 also underscores that weekends are substantially more enjoyable than weekdays even among retired people, which might reflect the importance of shared time with loved ones (who are in the workforce) or cultural cadences of errands and leisure that persist from working life into retirement—possibilities that raise evocative questions about what exactly makes time pleasant. Amid these shared daily rhythms, ethnoracial differences persist: on both weekdays and weekends, and for people of all employment levels, White people (solid lines) have lower daily unpleasantness than minoritized groups (dashed lines).

**Figure 8.**
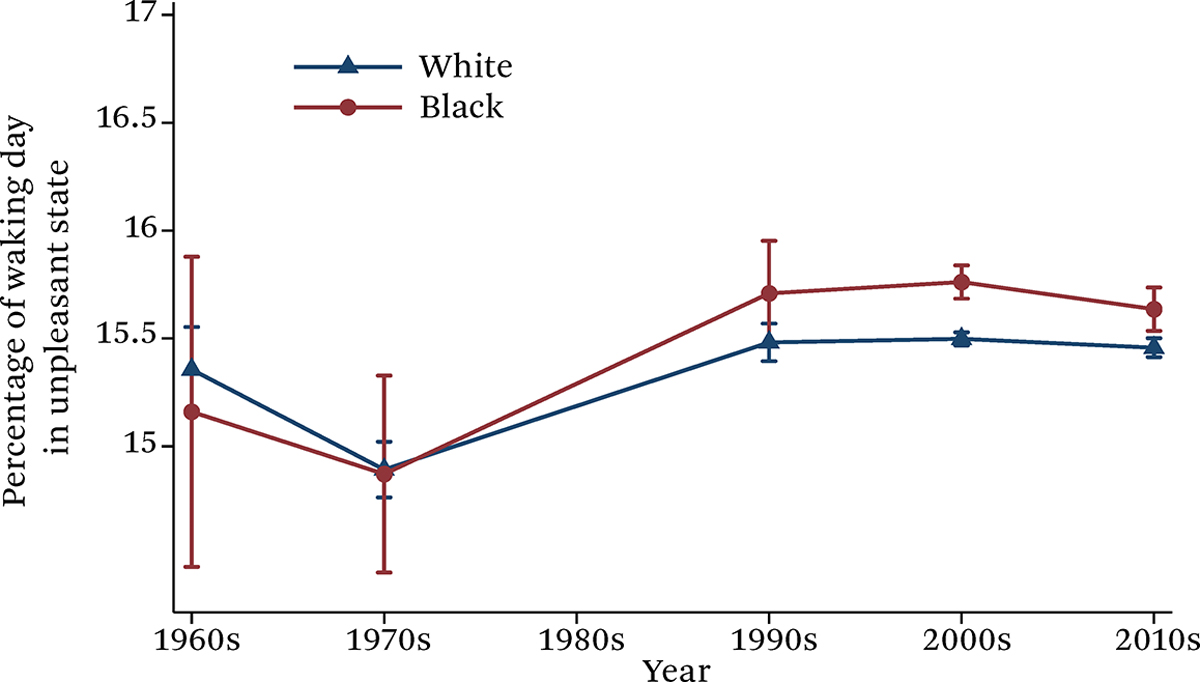
Historic Trends in Daily Unpleasantness *Source:* Authors’ calculations based on American Heritage Time Use Survey 1965–2018 (Fisher et al. 2018). *Note:* Adjusted predicted values and 95 percent confidence intervals from the American Heritage Time Use Survey 1965–2018. Predicted level of daily unpleasantness from ordinal least squares regressions of the proportion of the waking day spent in an unpleasant state. Estimates are adjusted for sex, age, age squared, educational attainment, employment status, marital status, any coresident children under age eighteen, census region, ten-year group, month, and day of week. Model includes an interaction term for ethnoracial group × ten-year group.

Figure 8 situates these disparities historically, presenting results for simplified racial groups. Though small sample sizes in early years make estimates imprecise, beginning in the 2000s, Black people’s daily lives are more unpleasant than those of White people. However, it is not possible to determine whether this is a true change over time or an artifact of the switch in historic datasets to the American Time Use Survey beginning in the 2000s and continuing through the 2010s.

## MODERATION BY SEX

Because research on daily unpleasantness has to date focused on sex differences (Krueger 2007), we also investigated whether sex moderated each of our findings. We confirmed that daily activities vary by sex and that most racial differences are consistent by sex. Patterns differed for a few activities, however. White women spend more time alone than White men, but this pattern is reversed for Black people, such that Black men spend more time alone than Black women. White men spend more time eating and drinking than White women. Additionally, Black and Hispanic men spend more time with friends than Black and Hispanic women, respectively. We also found that rates of reporting only positive emotions during daily activities did not vary by sex. Because we did not identify racial differences in the degree to which activities are rated as unpleasant, we did not test for sex moderation of those (nonexistent in the aggregate) racial differences. Finally, we found that White, Hispanic, and Asian men have about 1 percentage point higher daily unpleasantness than same-group women (online appendix E). After adjusting for sociodemographic covariates, differences are attenuated such that men have about 0.25 percentage point more daily unpleasantness than same-group women.

## DISCUSSION

Time is a fundamental social good with a finite limit, arguably the resource that people most wish to have more of or more control over. Recent years have seen increasing calls for research on the ethnoracial patterning of time use (Gee et al. 2019; Kwate 2017), and theoretical frameworks suggest that time use is foundational to racial inequities in outcomes such as population health (Colen et al. 2023). Yet little empirical research has explored patterns of time use across racial groups outside limited domains, such as patterns of full-time versus part-time employment. This gap is particularly surprising given extensive research on inequities in time use and daily activities by gender, another key characteristic of stratification in the United States. We argue that time use data represent an underused resource for understanding how daily life does—and does not—vary across subpopulations.

In this work, we analyze the time use patterns of more than two hundred thousand White, Black, Hispanic, and Asian people to understand whether and how patterns of daily activities vary by race and ethnicity. These differences can also create inequities in quality of life, although some may also reflect distinctive preferences that need not map neatly onto inequalities.

We found that some important aspects of time use show little variation across ethnoracial groups, highlighting how deeply constraining the core structures of time use are for all people. Moreover, despite the major social changes in the United States over the last half century, we find broad historical continuity in daily unpleasantness across the population: the portion of the day spent in unpleasant activities has hovered between roughly 15 and 15.5 percent of the waking day for both White and Black people from the 1960s to the 2010s. These relatively small differences can be consequential for experience, yet one might also have expected a larger shift over a period in which so many aspects of daily life have altered. Although the historical estimates are imprecise, we find similar continuity using robust data from the 2000s to 2010s. Our results offer suggestive evidence that population-level historical shifts have not been of much greater magnitude than the differences between racial groups’ unpleasant time today.

At the same time, other elements of time use show meaningful differences. The balance of time between more unpleasant obligatory activities and more pleasant leisure time varies across groups. Asian people spend the largest portion of their days in an unpleasant state, whereas White people spend the least time. Asian people’s disproportionate unpleasant time reflects that they both spend the most time in unpleasant activities (notably, paid work) and spend the least time in the most enjoyable activities (elective leisure). These findings are particularly notable given that Asian people have been less consistently included in research on daily life in the United States, suggesting the need for better understanding of their experiences. We also find variations within people’s unconstrained leisure time in the split between highly pleasant and affectively neutral leisure activities. White people spend the most time doing the most pleasant leisure activities, and Black people spend the most time doing affectively neutral activities such as watching television. However, these differences across groups are small relative to, for example, all ethnoracial groups’ difference in unpleasant time on weekdays versus weekends—even among people who are retired from the workforce.

Our analyses also uncovered several new stylized facts about how ethnoracial groups spend their time. For example, we showed that Black people spend the most time alone and Hispanic people spend the least alone. High levels of time alone are associated with higher risks of loneliness (Danvers et al. 2023). Yet solitude can also have psychological benefits (Long and Averill 2003) and too little time alone can also be its own source of stress (Buchanan, McFarlane, and Das 2024). Some research suggests that time alone is a “distinct ‘experiential niche’ having unique potentials and liabilities” (Larson 1990, 155), and other research suggests that time alone can dampen both positive and negative emotions (Nguyen, Ryan, and Deci 2018). Time alone might be broadly beneficial to the extent that people choose the contexts that they prefer, or deleterious if people have much more, or less, time alone than they would like. The benefits and drawbacks of substantial time alone also depend on the ready alternatives; for example, social support can reduce suicidal ideation, but negative social interactions can intensity it (Lincoln et al. 2012). The striking differences we uncovered in time alone merit further investigation in the context of other research finding that ethnoracial differences in social support vary with gender and life stage (Silverstein and Waite 1993).

The lack of research that includes all daily activities, and especially measures of how people feel during their activities, to systematically explore racial differences has left important aspects of daily life unexplored. Categories of activities that are prespecified by researchers may miss important nuances that appear when the categories are developed from respondent-provided information (in this case, about how often time spent doing various activities is unpleasant). For example, broad categories of free time cannot capture the distinctive trade-offs between elective leisure and neutral downtime.

These findings offer a new area for racial stratification research. Given long-standing inequities in educational attainment, income, and occupation across ethnoracial groups (Bloome and Western 2011; Wilson, Sakura-Lemessy, and West 1999), we expected to find the largest differences in time spent on activities related to market work (including main work, second jobs, commuting, education, and so on). We did identify differences in work-related time use, but differences in neutral downtime were equally large, and with whom people spent their time varied more across groups than their activities. These findings raise questions about the constraints and preferences that produce these patterns: Which structural conditions facilitate White people spending the most time on the most pleasant activities, even after accounting for socioeconomic resources? Do Black people spend more time alone and on neutral downtime activities by preference or due to constraints in the availability of people, money, energy, and neighborhood amenities? Which aspects of family networks lead Hispanic people to spend more time with young children and extended family than other ethnoracial groups? Do differences in occupation or self-employment explain why Asian people work more than other groups? Future research should decompose these differences to determine which factors create differences in daily activities across groups.

### Limitations

First, we acknowledge that applying contemporary measures of unpleasant activities historically requires the strong assumption that the experiential nature of daily activities has remained constant for the last half century, though this approach has been used (Krueger 2007).

Second, we caution against interpreting the u-index of a given activity as a measure of the average level of unpleasantness for that activity for everyone in the population. Unpleasantness is measured among people who do a given activity, and we apply it to other people who do the activity (the same target population). Yet people likely select out of activities they find most unpleasant, as feasible, so unpleasantness would likely be higher if the entire population were queried about all activities. Further, although we measured the unpleasantness of sixty-four unique daily activities, our activities may not have been specific enough to detect differences between the experiences of minoritized groups and White people. For example, witnessing police stops affects White and non-White adolescents differently (Jackson et al. 2021), and time with family may elicit different emotions across ethno-cultural contexts (Trieu 2016).

Third, information about emotions was not assessed for time spent sleeping or doing personal care activities, so we were not able to make estimates of total unpleasant time across the twenty-four-hour day. A large literature shows that non-White people sleep less and more poorly than White people (Billings et al. 2021). Understanding the degree to which people make trade-offs between sleep and other activities is important to comprehensively understanding time use, particularly time use during the waking day.

Fourth, the ATUS and AHTUS have very limited information on secondary activities done in conjunction with primary activities. Thus, we were not able to examine patterns of multitasking.

Fifth, research using time diaries faces recall bias, as respondents are asked to remember the previous day’s activities. Ecological momentary assessment using new technologies that capture daily activities in real time (Krueger et al. 2009) offer promising opportunities for future research in this area.

Finally, our ability to make historic conclusions was hampered by small sample sizes and limited to Black-White comparisons, given limited information on ethnoracial identity.

### Future Work Using Time Use Data to Study Daily Emotional Experiences

Population-level research on stress and daily experiences has often used surveys with broad questions about typical emotional states. In this work, we take the relatively unconventional approach of assessing daily emotional during specific daily activities using time diaries. We join other recent perspectives (Kwate 2017; Gee et al. 2019; Colen et al. 2023) that encourage using these data to explore key questions about daily life, including about how daily life may be structured differently across subpopulations. This approach offers several promising opportunities, including establishing theoretical and methodological practices for such work. We conclude by identifying some key remaining questions.

Theoretically, it is not clear how different measures of emotional experience are relevant to other outcomes. For example, we find that the most positive daily experiences are leisure activities, and White people spend the most time doing those activities. Black people spend the least time doing those activities but are also the most likely to report feeling only positive emotions during their daily activities. How do we reconcile these seemingly contradictory findings? And how do these findings inform research using other sources of reported data on emotions, like survey responses about typical emotional states? Which measures should we be using, and when?

Methodologically, time use data also provide a rich opportunity to explore the temporal component of daily emotions and provide a new lens for understanding the timing of daily activities. How do emotions from one activity bleed over into subsequent activities? How can enjoyable activities provide a buffer against challenging experiences? To what degree do people choose their daily activities based on their expected emotional tenor? Additionally, there is much opportunity to explore the analytic consequence of the use of the waking day (subtracting time spent on sleep and personal care) as the denominator in time use analyses. People adjust how much they sleep based on activities that they either need or wish to do. Sleep duration is both cause and consequence of mental health, and future work should more systematically integrate the study of sleep with the study of how time is spent during waking life to develop holistic measures of time use and well-being.

To conclude, we underscore that time diaries are a unique type of granular quantitative data that reveal life as it is experienced, day in and day out. Daily activities structure health and emotions; the contexts in which people spend their days shape relationships. Though many studies that describe the texture of daily life use qualitative data, time use data offer a compelling opportunity to explore how constraints and choices intersect in producing daily behaviors and experiences at a scale that facilitates subgroup comparisons. We hope that the results in this article will inspire much more exploration of these questions.

## Supplementary Material

Appendix A. Description of analytic samples

## Figures and Tables

**Table 1.** Datasets and Size of Analytic Samples

Survey	American Time Use Survey (ATUS)	ATUS Well-Being Module	American Historical Time Use Survey (AHTUS)

About dataset	Nationally representative time use study of the noninstitutionalized civilian population collected annually by the Bureau of Labor Statistics among a subset of participants in the Current Population Survey age fifteen and older.	Special module administered to a subset of ATUS participants (see left column) to collect information on emotional states during daily activities. Fielded in 2010, 2012, and 2013.	Harmonized series of time use surveys spanning 1930 to 2018. Includes ATUS data (see left column) from 2003 onward.
Used in	What People Do Each Day	How People Feel During Daily Activities	Historical and Contextual Variation
Total analytic sample size	210,586	37,088	155,891

*Source:* Authors’ tabulation.

*Note:* Sample sizes are weighted to be nationally representative.

## Footnotes

1. For example, between 1965 and 2012, the gender gap in time doing paid work declined by more than half and the gender gap in housework declined by about 75 percent among people age twenty-five to fifty-nine (Sayer 2016). Yet contemporary gender inequities remain: women still do 60 percent more housework (Bianchi et al. 2012) and more carework than men (Dukhovnov and Zagheni 2015).

2. See online appendix (https://www.rsfjournal.org/content/11/1/178/tab-supplemental).

3. We use the ethnoracial group names provided by ATUS.

4. Sleep and select personal care activities were not eligible for inclusion in the ATUS Well-Being Module.

5. Again, we borrow this terminology from Krueger (2007) but include a slightly different set of activities.

6. Despite the large time gap in applying the contemporary ATUS u-index ratings historically, contemporary ATUS affect data are similar to the limited affect measures available in historic time use datasets (Robinson 2013). Contemporary u-index measures have been applied historically in prior research (Krueger 2007).

7. We use waking day to refer to the daily activities for which unpleasantness was measured in ATUS/AHTUS, as in research on daily unpleasantness (Krueger 2007). However, this terminology is somewhat inaccurate, given that the so-called waking day excludes both time spent sleeping and time doing personal care activities.
