# Supplementary material for: How Ethnoracial Groups Spend Their Time
Source: RSF. Author manuscript; Available in PMC 2026 Jan 1. (PMC11887659; doi:10.7758/rsf.2025.11.1.09)
Supplement: Appendix A. Description of analytic samples [file NIHMS2053736-supplement-Appendix_A__Description_of_analytic_samples.pdf]

Online Appendix A. Description of analytic samples
(authors' calculations of means or percentages; estimates are weighted to be nationally representative)

**Survey**	**American Time Use Study (ATUS)**	**ATUS Well-being Module**	**American Historical Time Use Study (AHTUS)**
**Total analytic N**	210,586	37,088	155,891
**N by year**	2003, N = 20,720	2010, N = 13,260	1965, N = 2,021
	2004, N = 13,973	2012, N = 12,443	1975, N = 4,546
	2005, N = 13,038	2013, N = 11,385	1993, N = 7,471
	2006, N = 12,943		1995, N = 1,189
	2007, N = 12,248		1998, N = 2,337
	2008, N = 12,723		2003, N = 19,484
	2009, N = 13,133		2004, N = 13,165
	2010, N = 13,260		2005, N = 12,257
	2011, N = 12,479		2006, N = 12,056
	2012, N = 12,443		2007, N = 11,478
	2013, N = 11,385		2008, N = 11,956
	2014, N = 11,592		2009, N = 12,451
	2015, N = 10,905		2010, N = 12,524
	2016, N = 10,493		2011, N = 11,853
	2017, N = 10,223		2012, N = 11,850
	2018, N = 9,593		2018, N = 9,253
	2019, N = 9,435		
**Demographic characteristics**			
Ethnoracial group (ATUS)			
White, non-Hispanic	68 percent	67 percent	N/A
Black, non-Hispanic	12 percent	12 percent	N/A
Hispanic	15 percent	15 percent	N/A
Asian, non-Hispanic	4 percent	4 percent	N/A
Other race/ethnicity	2 percent	2 percent	N/A
Ethnoracial group (AHTUS)			
White	N/A	N/A	81 percent
Black	N/A	N/A	13 percent
Other	N/A	N/A	5 percent
Sex			
Male	48 percent	48 percent	48 percent
Female	52 percent	52 percent	52 percent
Age	45 years	45 years	47 years
Educational attainment			
Less than high school	17 percent	17 percent	11 percent
High school graduate or GED	29 percent	29 percent	28 percent
Some college	25 percent	25 percent	20 percent
College graduate	18 percent	19 percent	30 percent
Any post-graduate education	10 percent	10 percent	12 percent
Employment status (ATUS)			
Employed full time	49 percent	47 percent	N/A

Employed part time	14 percent	14 percent	N/A
Not working – not retired	22 percent	25 percent	N/A
Not working – retired	15 percent	14 percent	N/A
Employment status (AHTUS)			
Employed full time	N/A	N/A	54 percent
Employed part time	N/A	N/A	13 percent
Not working	N/A	N/A	33 percent
Nativity			
Native born	86 percent	85 percent	N/A
Foreign born	14 percent	15 percent	N/A
Martial status			
Married	53 percent	52 percent	N/A
Widowed, separated, divorced	17 percent	17 percent	N/A
Unmarried	30 percent	30 percent	N/A
Marital status (AHUS)			
Married	N/A	N/A	55 percent
Unmarried	N/A	N/A	45 percent
Any coresident children	40 percent	40 percent	44 percent
Urban/rural			
Urban	83 percent	84 percent	N/A
Rural	17 percent	16 percent	N/A
Census region			
Northeast	18 percent	18 percent	17 percent
Midwest	24 percent	24 percent	25 percent
South	36 percent	36 percent	36 percent
West	22 percent	22 percent	22 percent
Month of time diary			
January	9 percent	9 percent	10 percent
February	7 percent	7 percent	8 percent
March	8 percent	8 percent	9 percent
April	8 percent	9 percent	8 percent
May	8 percent	8 percent	8 percent
June	8 percent	8 percent	8 percent
July	8 percent	9 percent	8 percent
August	9 percent	8 percent	8 percent
September	8 percent	8 percent	8 percent
October	9 percent	8 percent	8 percent
November	8 percent	9 percent	8 percent
December	9 percent	9 percent	8 percent
Day of week of time diary			
Monday	14 percent	14 percent	14 percent
Tuesday	14 percent	14 percent	14 percent
Wednesday	14 percent	14 percent	14 percent
Thursday	14 percent	14 percent	14 percent
Friday	14 percent	14 percent	14 percent
Saturday	14 percent	14 percent	14 percent
Sunday	14 percent	14 percent	14 percent

Note: Percentages may not sum to 100 due to rounding.

Online Appendix B. Portion of neutral downtime spent watching television/videos
(unadjusted and adjusted[1] predicted values from the
American Time Use Survey, 2003-2019)

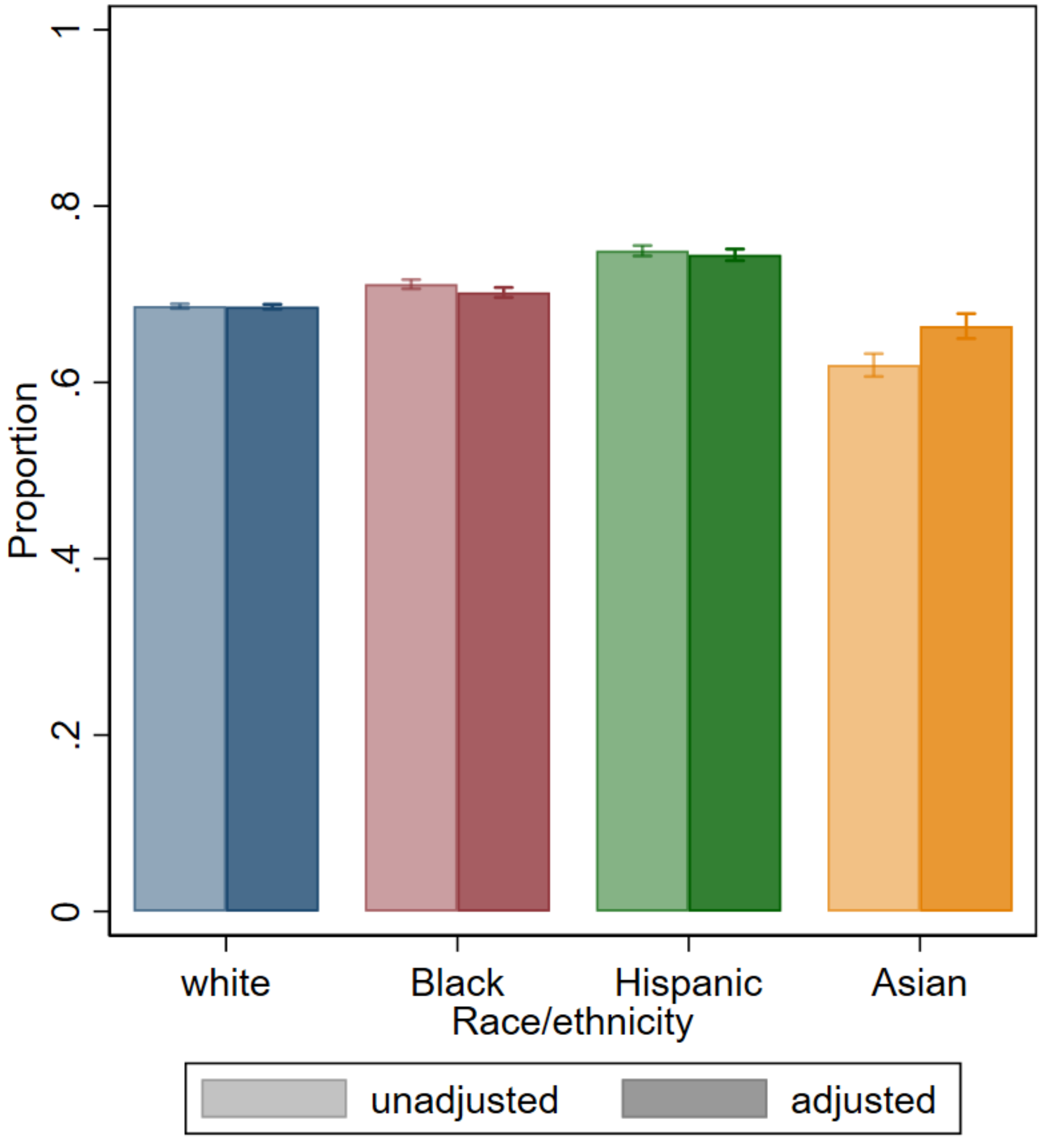

[1] Authors' calculations. Adjusted estimates are adjusted for age, age squared, educational attainment, employment status, nativity, marital status, any coresident children under age 18, urban/rural residence, Census region, ten-year group, month, and day of week.

Online Appendix C. Ethnoracial differences in ratio of time spent in daily activities to white people's time in activity

(unadjusted and adjusted[2] predicted values and 95% confidence intervals from the American Time Use Survey 2003-2019)

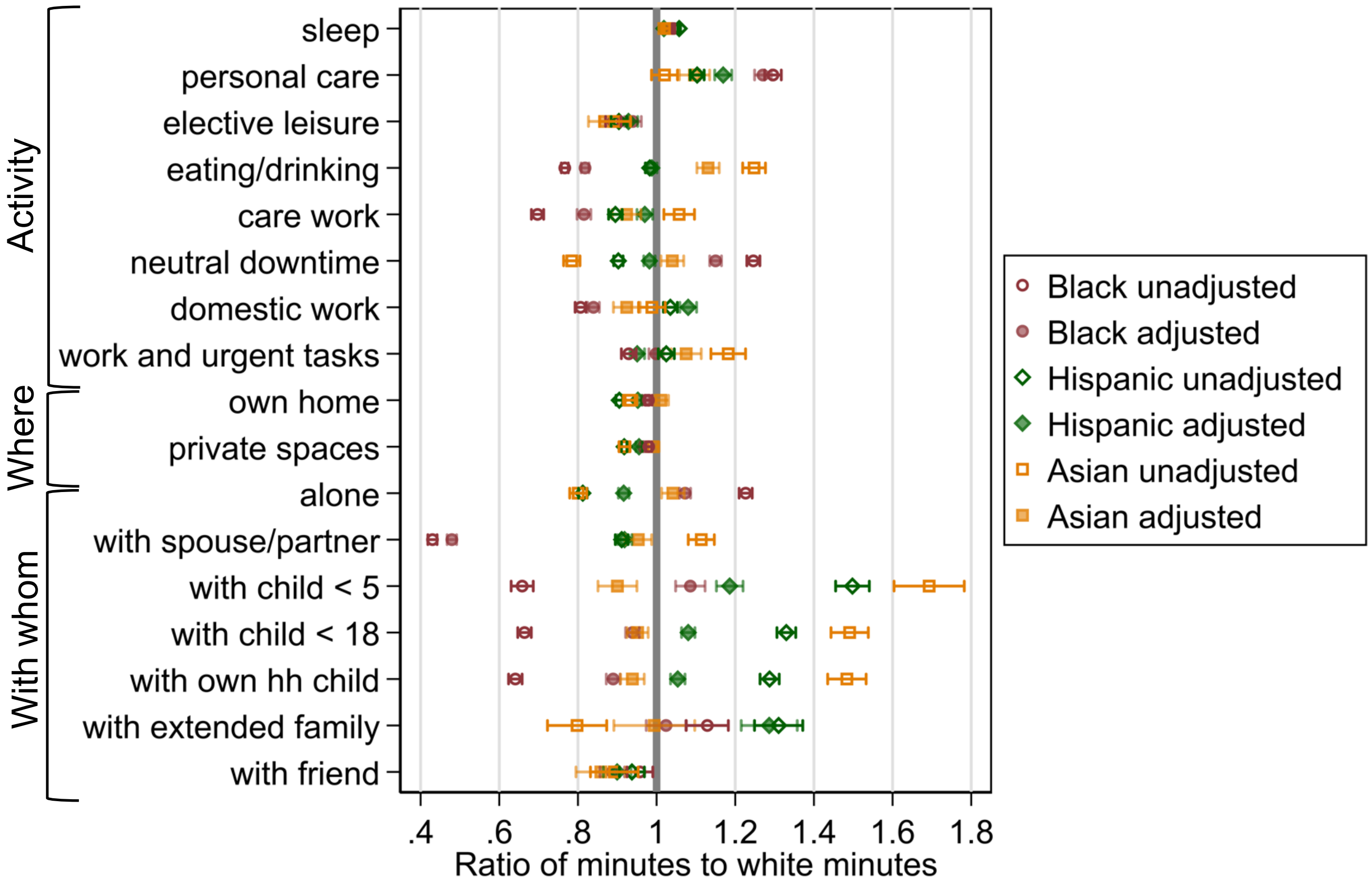

---

[2] Authors' calculations of marginal effects from count models of time spent on daily activities: negative binomial models of number of minutes spent on sleep, personal care, elective leisure, eating and drinking, neutral downtime, domestic work and errands, at home, in private spaces, alone, with extended family, and with friends and zero-inflated negative binomial models to model the number of minutes spent on work and urgent tasks, with spouse/partner, with children under age 5, with children under age 18, and with a coresident child. Estimates are adjusted for sex, age, age squared, educational attainment, employment status, nativity, marital status, any coresident children under age 18, urban/rural residence, Census region, ten-year group, month, and day of week.

Online Appendix D. Ethnoracial similarity in daily unpleasantness of exemplar activities
(estimates and 95% confidence intervals[3] from the
American Time Use Survey Well-Being Model, 2010, 2012, 2013)

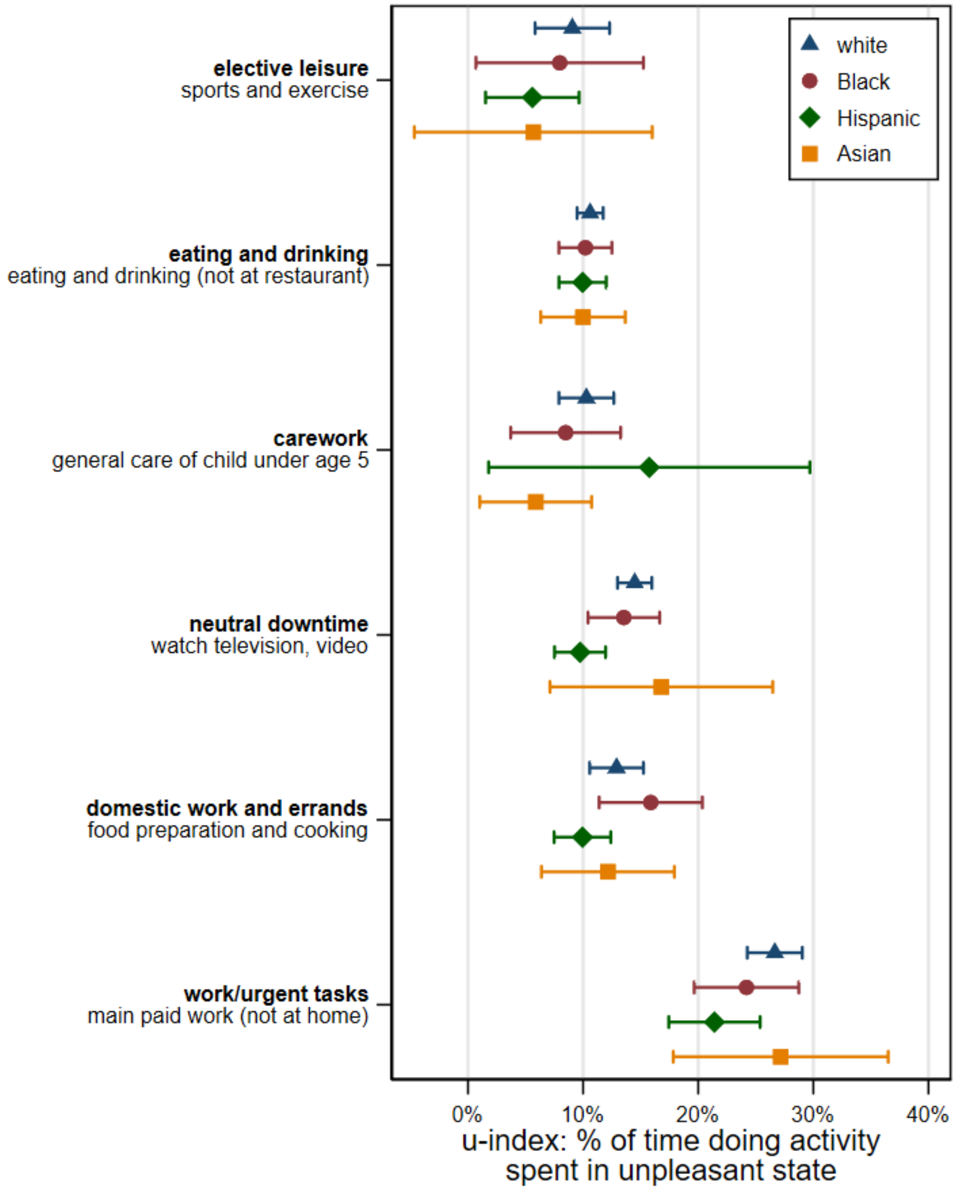

[3] Authors' calculations of average u-index values by ethnoracial group across 160 race-specific u-index calculations (using 160 replicate weights). Confidence intervals represent the uncertainty in the u-index estimate from using 160 different replicate weights.

Online Appendix E. Daily unpleasantness by sex

(unadjusted and adjusted[4] predicted values and 95% confidence intervals from the American Time Use Survey, 2003-2019)

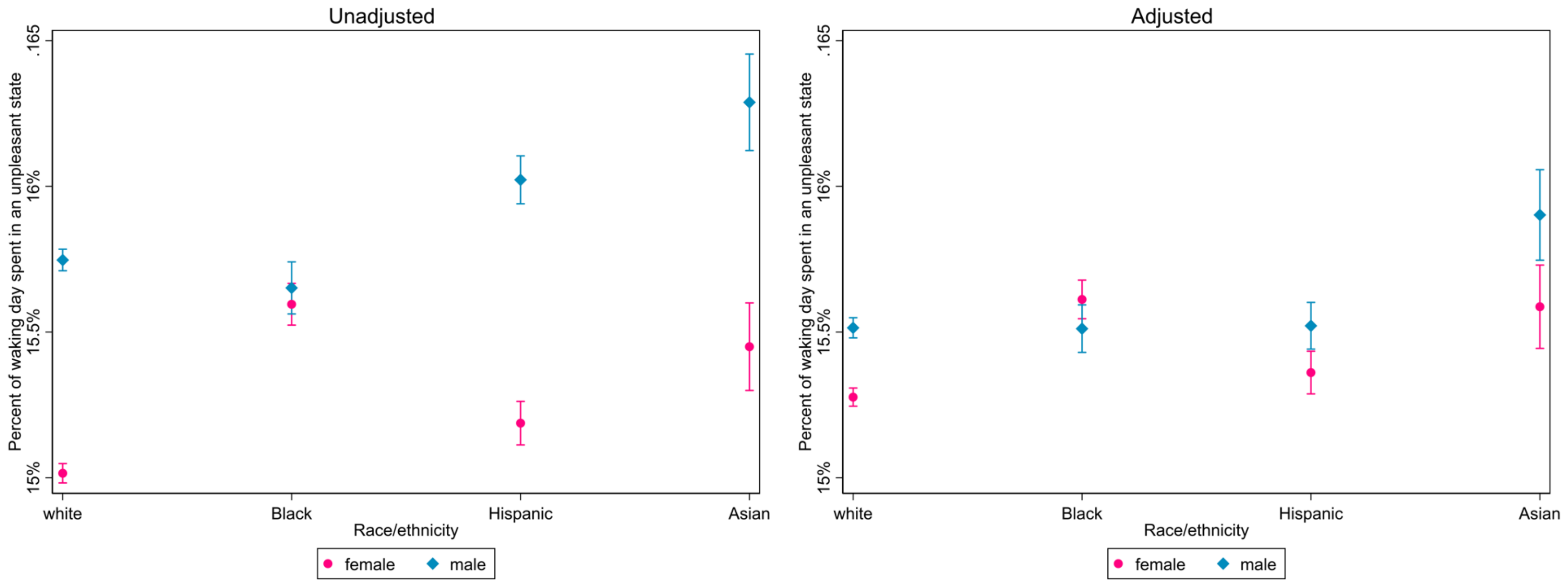

[4] Authors' calculations of predicted level of daily unpleasantness from ordinal least squares regressions of the proportion of the waking day spent in an unpleasant state. Models comparing include an interaction term for sex * ethnoracial group. Estimates are adjusted for age, age squared, educational attainment, employment status, nativity, marital status, any coresident children under age 18, urban/rural residence, Census region, ten-year group, month, and day of week.
